# Dynamic consent, communication and return of results in large-scale health data reuse: Survey of public preferences

**DOI:** 10.1177/20552076231190997

**Published:** 2023-08-16

**Authors:** Sam HA Muller, Ghislaine JMW van Thiel, Menno Mostert, Johannes JM van Delden

**Affiliations:** 168086 Julius Center for Health Sciences and Primary Care, University Medical Center Utrecht, Utrecht, The Netherlands

**Keywords:** Data-intensive health research, governance, health informatics, research ethics, dynamic consent, return of results

## Abstract

Dynamic consent forms a comprehensive, tailored approach for interacting with research participants. We conducted a survey study to inquire how research participants evaluate the elements of consent, information provision, communication and return of results within dynamic consent in a hypothetical health data reuse scenario. We distributed a digital questionnaire among a purposive sample of patient panel members. Data were analysed using descriptive and nonparametric inferential statistics. Respondents favoured the potential to manage changing consent preferences over time. There was much agreement between people favouring closer and more specific control over data reuse approval and those in favour of broader approval, facilitated by an opt-out system or an independent data reuse committee. People want to receive more information about reuse, outcomes and return of results. Respondents supported an interactive model of research participation, welcoming regular, diverse and interactive forms of communication, like a digital communication platform. Approval for reuse and providing meaningful information, including meaningful return of results, are intricately related to facilitating better communication. Respondents favoured return of actionable research results. These findings emphasize the potential of dynamic consent for enabling participants to maintain control over *how* their data are being used for *which* purposes by *whom*. Allowing different options to shape a dynamic consent interface in health data reuse in a personalized manner is pivotal to accommodate plurality in a flexible though robust manner. Interaction via dynamic consent enables participants to tailor the elements of participation they deem relevant to their own preferences, engaging diverse perspectives, interests and preferences.

## Introduction

The wide scope and large scale of research reusing existing health data is unprecedented and yields great potential.^1–3^ Yet sharing, linking and reusing health data from different sources pose important ethical and legal challenges.^1,4–6^ These challenges urge us to reconsider how health data reuse can be governed responsibly.

Effective authorization of data reuse is paramount to ensure health data research that is not only ethically sound but also legitimate and sustainable. At the same time, retaining specific informed consent as the gold standard for authorizing large-scale sharing and reuse of health data has become increasingly less feasible and desirable.^4^ In the first place, some have suggested that consent may not always be necessary as a basis for authorizing reuse for forms of large-scale health data research.^7–9^ More importantly, large-scale health data research reveals the so-called ‘consent or anonymize’ paradigm, which oftentimes leads to overemphasis in governance on the moment of initial consent, disregarding other concerns of data-contributing participants.^8^ Within the consent or anonymize paradigm, possibilities to adapt specific informed consent requirements as well as the alternative of de-identifying personal health data in the form of anonymization are limited. In addition, fragmented implementation of research exemptions in national law and prespecified conditions like purpose limitations hinder international research collaboration premised on data sharing.^1,4^ Furthermore, the extent to which it is possible to seek valid and meaningful consent for unforeseeable future use is called into question in the literature as a realistic way forward, given the increasing scale and scope of scientific research relying on reuse of health-related data.^4,10,11^ In sum, the static one-off nature of specific informed consent does not pair well with demands for flexible and smooth use of large amounts of varied data.^6,10,12,13^

Alternatives like blanket consent,^14^ meta consent,^15^ tiered consent,^16^ broad consent^17,18^ and dynamic consent^19–23^ have been formulated to amend these shortcomings. Blanket consent refers to agreeing to health data reuse without any restrictions, covering in particular future research uses.^14^ Meta consent focuses on how and when people would like to provide consent in the future.^15^ Tiered and broad consent however specify consent for specific categories, tiers, scopes or goals of research.^16,17,24^ Broad consent can be understood as giving consent for governance by certain institutions for a broad but still sufficiently specified research purpose.^2,4,25,26^ Since such consent can only be meaningfully given within circumscribed governance contexts, straightforward responsible governance forms a condition for broad consent. For instance, conditions for oversight and sanctioning of possible misuse are pertinent elements for such approval to be given.^2,27^

Dynamic consent proposes a digital interface for research participants to continuously renew and alter their consent preferences, depending on the research as they see fit.^4,21,22,28^ Dynamic consent enables multiple forms of authorization, ranging from specific and broad opt-in consent to an opt-out system.^23^ A dynamic, tailored interface stimulates engagement by going beyond consent as the main way of participating and connecting with health data research.^29,30^ It aims for research participants to enhance their control over what is being done with their health data, as has been advocated under the guise of personal data cooperatives.^31,32^ In addition to consent, a dynamic consent interface entails two-way communication, providing information and the return of relevant results to participants.^19,22,28,30,33^ To enable clear-cut and responsible governance, dynamic consent complements the need for authorization of data reuse by seeking meaningful participation and involvement in health data research.

Empirical research to further understand participants’ attitudes about the elements of a dynamic consent interface has been undertaken, highlighting in particular the importance of communication and engagement in dynamic consent.^19,29,34^ However, having been developed in response to challenges to consent to future research in biobanking,^19^ much research has focused on participants’ attitudes in the context of biobanking.^18,20,30,35–37^ Moreover, the empirical literature focusing on stakeholder perspectives on dynamic consent beyond biobanking^21,38–45^ mainly comprises qualitative studies and case evaluations that are strongly context-bound, which impedes generalization.^29^

Research into preferences of the public for sharing health data across different contexts highlighted enabling research participants to be informed about their data being shared, having a review process to oversee the sharing and use of data and the ability to opt out from sharing data as important governance mechanisms.^46^ In line with this, the purpose of this study is to inquire about people's preferences for consent, communication and information provision as well as the return of results for a hypothetical dynamic consent interface for large-scale health data research. These preferences can be used to inform and enable the shaping of governance arrangements by means of dynamic consent in the form of policies and measures for large-scale health data research in which reuse plays an important role.

We address four themes: (a) consent for health data reuse, (b) communication and information provision, (c) return of results and (d) oversight and sanctions for health data misuse.

## Materials and methods

### Aim and design

Reporting of this survey study conforms to the Checklist for Reporting of Survey Studies (CROSS).^47^ The aim of this cross-sectional survey was to gain greater insight into which preferences research participants have for each of the elements of dynamic consent for health research in which data are reused on a large scale. Moreover, we wanted to gain a better understanding of how their preferences for consent, communication and information provision, return of results and oversight and sanctions related to each other in the case of reuse of their data for scientific health research. The final questionnaire version was constructed by building on theoretical and empirical reviews of dynamic consent.^19,22,28,29,48^ We specified health data for reuse as data that could stem from both care and research and would be shared pseudonymously. Questions were illustrated by providing various examples of health data reuse in relatable, real-life situations.

The first and second versions of the questionnaire were reviewed and pilot tested with two panel members that were demographically similar to the sample population and one patient and public involvement and engagement (PPIE) professional of the University Medical Center Utrecht's patient panel in the Netherlands. Subsequently, minor changes were made to question phrasing to improve understandability and connection with the patient perspective. Pretesting participants were excluded from filling in the questionnaire. The final questionnaire comprised five sections (consent, communication and information, misuse, return of results and background information) consisting of 21 questions, which took respondents approximately 12 minutes to fill in. The questionnaire was translated and distributed in Dutch. The questionnaire can be found in the Supplemental Information.

We used 5-point scale Likert-item questions and multiple-choice questions. Respondents were also asked to explain their answers or to provide alternative answers in open text fields. Prior to the start of the questionnaire, informed consent was given by respondents for the use of their answers for scientific research. Duplicate entries were avoided by using cookies expiring after 6 months, preventing users from accessing the survey twice.

### Ethical considerations

Approval from an ethics committee was not necessary for this type of unobtrusive, nonmedical scientific research. Under Dutch law, this research is exempt from review by a medical research ethics committee (Medical Research Involving Human Subjects Act (WMO); Central Committee on Research Involving Human Subjects). Participants gave their informed consent for the use of their answers for scientific research prior to the start of the questionnaire.

### Setting

The survey was conducted online via digital distribution by purposive sampling among members of the University Medical Center Utrecht's patient panel. The patient panel collects patient experiences, expectations and desires to improve health care and research. It comprises people that feel involved with the University Medical Center Utrecht and volunteer to be consulted about topics such as digitalization, patient safety, service, care and treatment. The survey was distributed using the panel's online communication and administration platform. Respondents were contacted as part of the regular email communication of the patient panel with a call to participate in the survey. A targeted reminder was sent after 2 weeks to nonresponding panel members without revealing their identity to the research team, which was facilitated by the panel's own online communication and administration platform. The survey was administered using the Qualtrics XM survey tool. The inclusion criteria were the age of 18+ years and being a member of the panel, which includes being a patient or being otherwise involved in the University Medical Center Utrecht. Participation was voluntary and without incentives. The survey was accessible from 9 January 2022 to 31 February 2022.

### Analysis

Both complete and incomplete questionnaires were analysed. The resulting data were stratified by the demographic variables of age, education level and gender. Analysis comprised descriptive statistics and exploring interrelations within and between thematized variables by subsequent statistical tests. Moreover, explanations or alternative suggestions provided in the open text fields were used to inform interpretation of the quantitative findings. Data were analysed using IBM SPSS Statistics, version 26 (IBM Corp., Armonk, NY). For Likert-item ordinal variables, we reported descriptive statistics including response percentages for each category, the median (*Md*) and the interquartile range (*IQR*). For multiple-choice categorical variables, we reported frequencies and percentages for each category as well as the mode (*Mo*). For the descriptive statistics, we reported valid percentages.

We compared groups and tested for associations between preferences for consent, communication and information provision and return of results by using inferential statistics. Since the assumptions underlying parametric statistics were violated, nonparametric *χ*^2^ tests of independence, Mann–Whitney *U* tests, Kruskal–Wallis tests and Spearman rank-order correlations were used.^49^ Moreover, nonparametric statistics were appropriate due to the ordinal and categorical measurement levels of the variables.^50–53^

An alpha (*α*) level of .05 was employed to determine significance for all statistical tests. All tests we used were two-tailed. The underlying assumptions were met for all (nonparametric) tests reported. Missing data have been assumed ignorable since missing data were diffuse, less than 5% of data were missing for all variables and specific missing data patterns were not apparent. We used customary pairwise deletion of cases to treat missing data in the nonparametric statistical tests, which is a robust method for large sample sizes with small amounts of scattered missing data.^54,55^ Sample sizes varied slightly across different tests as a result. Dependent variables were dummy coded to infer the directions of associations for *χ*^2^ tests of independence.

## Results

A total of 902 out of the 1928 members of the patient panel took part in the survey, resulting in a response rate of 46.8%. In general, 64.6% (*Md *= 5, *IQR *= 4,5) of the respondents strongly favoured reuse of their health data for scientific research. Only 1.4% indicated they somehow opposed this. See Table 1 for a detailed overview of the background characteristics.

**Table 1.** Frequencies of background characteristics (*n* = 902).

Variables		Results, *n* (%)^a^
Gender^b^		
	Female	410 (47.3)
	Male	455 (52.5)
	Other	2 (0.2)
Age range (years)^c^		
	18–30	14 (1.6)
	31–40	55 (6.4)
	41–50	74 (8.5)
	51–60	185 (21.4)
	61–70	289 (33.4)
	≥71	249 (28.8)
Country of residence^d,e^		
	Netherlands	859 (99.7)
Education level^f^		
	Primary school	7 (0.8)
	Secondary/high school	95 (11)
	Initial vocational education	42 (4.9)
	Secondary vocational education	164 (19)
	Higher education	341 (39.4)
	Academic education	207 (23.9)
	Other	9 (1)
Identification as a patient^g^		
	Yes	614 (71.5)
	No	245 (28.5)

^a^ Percentages given are valid percentages; *n* varies per variable.

^b^ *n* = 867.

^c^ *n* = 866.

^d^ Countries with a percentage >0.5% are shown.

^e^ *n* = 862.

^f^ *n* = 865.

^g^ *n* = 859.

In their explanations, respondents expressed that they attached great value to contributing to progress in scientific research. Many explanations highlighted altruistic reasons or motivations for sharing health data. As such, one respondent explained: ‘To me, what matters most when making my data available for research is that the common good is at the forefront, so that better treatments can be developed in the future for other patients’.

### Consent for reuse of health data

A total of 61.4% (*Mo *= 1) of the respondents *agreed* to give consent for reuse of their health data on the condition of knowing the research question, and 81.9% (*Mo *= 1) *agreed* to consent to a broad range of research questions. More importantly, 69.8% (*Mo *= 2) *preferred* consenting to health data reuse for a broad range of research questions, whereas 30.2% *preferred* knowing the specific research question before giving consent. Additionally, 25.7% (*Md *= 3, *IQR *= 2,5) *strongly favoured* to approve reuse of their data by new research by re-consenting. However, 28.1% (*Md *= 3, *IQR *= 2,4) of the respondents were *neutral* about an opt-out system in which one can object to data reuse, which allows reuse without re-consent. Similarly, 24.7% (*Md *= 3, *IQR *= 2,4) were *neutral* about approval by an independent committee for reuse of new research.

A total of 43% (*Md *= 4, *IQR *= 3,5) of the respondents *strongly favoured* experts like scientists, lawyers and ethicists as members of such a reuse approval committee. However, 27.3% (*Md *= 3, *IQR *= 2,5) strongly supported including patients’ and citizens’ representatives as committee members as well. Lastly, 32.8% of the respondents (*Md *= 6, *IQR *= 5,8) *did not care* how frequently their consent was sought. See Tables S1 and S2 in the Supplemental Information for a detailed overview of the descriptive results.

Respondents mentioned efficiency and effectiveness when consenting to sharing their data, to contribute as much for public benefit as possible, both for the sake of researchers, contributors of data and patients and for the improvement of health care in general: ‘Consent requests for each specific research question would mean a massive amount of paperwork. That would hold back researchers to use these data, or it would lead to me as patient having to (digitally) “sign” to give permission. That does not seem convenient’. Respondents explained that retaining a sense of control was important: ‘It gives a feeling of control, involvement and voice about the use of my data. Each time, “the patient” can make a conscious choice, depending on the specific research question’. Moreover, the role of *trust* within familiar contexts and the notion of mutual loyalty when giving consent were often emphasized: ‘Especially important for my decision is who (which institution, physician, department) is asking for consent: I want to have a “feeling” of trustworthiness of that person or institution, particularly concerning privacy and security’. As this quote highlights, there was an important relation with having insight by accurate, specific and appropriate information provision and communication about privacy and security measures as well as data sharing and research practices and goals.

Older respondents more frequently preferred an opt-out system for approving data reuse (*ρ *=* *−0.073, *P *= .035, *n *= 836). Respondents who preferred specific consent supported re-consent for health data reuse by new research to a significantly greater extent than those who preferred broad consent. Similarly, people who preferred broad consent favoured a committee for approving reuse more (see Table 2). Contrary to supporters of specific consent, adherents of broad consent favoured expert members of an independent reuse approval committee (see Table 2).

**Table 2.** Association between preference for consent specificity and modes of approving health data reuse, assessed using a Mann–Whitney *U* test.

Variable		Median	Mean rank	*U*	*z*	*P* value
**I want to re-consent before new research can reuse my health data. (*n* = 892)**						
	Specific (*n* = 270)	5	626	35,508	−14.02	<.001
	Broad (*n* = 622)	3	269			
**I can object to (opt out of) reuse of my health data by new research; I will not be asked to re-consent. (*n* = 870)**						
	Specific (*n* = 259)	3	430	77,640	−0.45	.653
	Broad (*n* = 611)	3	438			
**An independent committee approves whether new research can reuse my health data. (*n* = 864)**						
	Specific (*n* = 255)	3	370	61,738	−4.87	<.001
	Broad (*n* = 609)	3	459			
***An independent committee should comprise …***						
**Mainly experts like scientists, lawyers and ethicists (*n* = 864)**						
	Specific (*n* = 257)	4	369	61,666	−5.14	<.001
	Broad (*n* = 607)	4	459			
**Next to experts, (representatives of) patients and citizens (*n* = 859)**						
	Specific (*n* = 255)	4	428	76,555	−0.14	.888
	Broad (*n* = 604)	3	431			

Furthermore, re-consent was most strongly associated with preference for a committee made up of patients, citizens and experts (*ρ *=* *0.086, *P *= .011, *n *= 857). However, respondents who favoured an opt-out system (*ρ *=* *0.083, *P *= .016, *n *= 846) and a committee for approving reuse (*ρ *=* *0.258, *P *< .001, *n *= 839) most strongly preferred an expert-only committee.

In comparison to respondents who preferred broad consent (*Md *= 3, *n *= 628), those who preferred specific consent (*Md *= 4, *n *= 271) preferred doing so more frequently (*U *= 74,512, *z *= −3.05, *P *= .002). Additionally, people who preferred re-consent also wanted to do so more often (*ρ *=* *0.091, *P *= .007, *n *= 892).

### Communication and information regarding health data reuse

A total of 71.3% of the respondents wanted to be informed about reuse of their health data (*Mo *= 1). On top of this, 83.9% (*Mo *= 1) wanted to know of the scientific results accomplished. Additionally, 31.5% (*Md *= 4, *IQR *= 3,4) *somewhat favoured* being informed by a regularly updated website with information about the research reusing their data. Furthermore, 46.5% (*Md *= 4, *IQR *= 4,5) of the respondents somewhat favoured receiving newsletters via email with summaries and updates about the research. Lastly, a digital profile to tailor communication to one's personal preferences and interact with researchers and other participants was somewhat favoured by 36.9% (*Md *= 4, *IQR *= 3,5). See Table S3 in the Supplemental Information for a detailed overview of the descriptive results.

Age (*χ*^2^_10_ (*n = *866) = 22.7, *P *= .012, *φ*_c_*_ _* = 0.115) and gender (*χ*^2^_2_ (*n = *865) = 20.5, *P *< .001, *φ*_c_*_ _* = 0.154) differences were associated with the desire to be informed about health data reuse. People that wanted to receive such information preferred email newsletters and a digital communication profile more than those that did not. Respondents who did not consider it important to be informed supported communication by a website with information about research more. See Table 3 for an overview.

**Table 3.** Association between desire to be informed about health data reuse and preference for modes of information provision, assessed using a Mann–Whitney *U* test.

Variable		Median	Mean rank	*U*	*z*	*P* value
**I want to be informed by means of a website with up-to-date information about research reusing my data. (*n* = 818)**						
	Yes (*n* = 625)	3	399	53,953	−2.29	.022
	No (*n* = 193)	4	442			
**I want to be informed by means of email newsletters containing short summaries and updates about research using my data. (*n* = 825)**						
	Yes (*n* = 631)	4	438	45,672	−5.81	<.001
	No (*n* = 194)	4	333			
**I want to be informed by means of a digital profile allowing me to tailor communication to my personal preferences. (*n* = 811)**						
	Yes (*n* = 615)	4	429	46,205	−5.2	<.001
	No (*n* = 196)	4	334			

Re-consent was associated with the desire to receive information about health data reuse (*χ*^2^_8_ (*n = *894) = 159.6, *P *< .001, *φ*_c_*_ _* = 0.299). Desire to be informed was also associated with preference for an opt-out system (*χ*^2^_8_ (*n = *872) = 21, *P *= .007, *φ*_c_*_ _* = 0.110) and a reuse approval committee (*χ*^2^_8_ (*n = *866) = 46.8, *P *< .001, *φ*_c_*_ _* = 0.164). Respondents who preferred re-consent were most interested in receiving information about reuse (89.7%). However, communication of scientific results was not significantly associated with support for re-consent (*χ*^2^_8_ (*n = *893) = 11.3, *P *= .186, *φ*_c_*_ _* = 0.079) or support for an opt-out system (*χ*^2^_8_ (*n = *871) = 3.3, *P *= .921, *φ*_c_*_ _* = 0.044). Desire for scientific results communication was significantly associated with preference for a reuse approval committee (*χ*^2^_8_ (*n = *866) = 18.5, *P *= .018, *φ*_c_*_ _* = 0.103). This kind of information was desired by 85.3% of those who favoured such a committee.

Several methods for approving data reuse were associated with ways to inform respondents (see Table 4). In particular, support for re-consent was associated with a higher preference for a digital communication profile. Support for an opt-out system was associated with a greater desire for communication by a website with information about reuse.

**Table 4.** Spearman rank-order correlations (*ρ*) between preference for modes of approving data reuse and modes of information provision.

Variable		1. I want to re-consent before new research can reuse my health data.	2. I can object to (opt out of) reuse of my health data by new research; I will not be asked to re-consent.	3. An independent committee approves whether new research can reuse my health data.	4. I want to be informed by means of a website with up-to-date information about research reusing my data.	5. I want to be informed by means of email newsletters containing short summaries and updates about research using my data.	6. I want to be informed by means of a digital profile allowing me to tailor communication to my personal preferences.
**1. I want to re-consent before new research can reuse my health data.**							
	*ρ*	1	0.129	−0.076	−0.140	0.014	0.139
	*P* value	—^a^	<.001	<.001	<.001	.687	<.001
**2. I can object to (opt out of) reuse of my health data by new research; I will not be asked to re-consent.**							
	*Ρ*	0.129	1	0.245	0.094	0.017	0.064
	*P* value	<.001	—^a^	<.001	.007	.617	.065
**3. An independent committee approves whether new research can reuse my health data.**							
	*ρ*	−0.076	0.245	1	0.114	0.046	0.006
	*P* value	<.001	<.001	—^a^	.001	.186	.854
**4. I want to be informed by means of a website with up-to-date information about research reusing my data.**							
	*ρ*	−0.140	0.094	0.114	1	0.251	0.125
	*P* value	<.001	.007	.001	—^a^	<.001	<.001
**5. I want to be informed by means of email newsletters containing short summaries and updates about research using my data.**							
	*ρ*	0.014	0.017	0.046	0.251	1	0.362
	*P* value	.687	.617	.186	<.001	—^a^	<.001
**6. I want to be informed by means of a digital profile allowing me to tailor communication to my personal preferences.**							
	*ρ*	0.139	0.064	0.006	0.125	0.362	1
	*P* value	<.001	.065	.854	<.001	<.001	—^a^

^a^ Not applicable.

Respondents frequently mentioned interest in knowing for which purposes, by whom and how one's data are being used for research as important motivators for their preferences for information provision and communication. They explained that adequate information and insight made them feel more in control: ‘I want to retain control and keep track of the amount of times my data are used. I also want to know what my data are used for’.

### Return of results in health data reuse

A total of 78.8% (*Md *= 5, *IQR *= 5,5) of the respondents expressed a strong desire for return of results from reuse of their data. Receiving actionable results was *highly important* to 81.4% (*Md *= 5, *IQR *= 4,5). Additionally, 55.3% (*Md *= 5, *IQR *= 3,5) of the respondents indicated that results flagged as abnormal and possibly relevant to health were of *high importance* as well. Regardless of importance to their health, 29.3% (*Md *= 4, *IQR *= 3,5) considered the return of all results classified as abnormal *neither important nor unimportant*. In contrast, 59.5% (*Md *= 5, *IQR *= 4,5) of the respondents regarded return of information about a potentially dangerous genetic mutation or reproductive risk as *highly important*. Complete access to all information recorded by their data being reused was considered highly important by 44.3% (*Md *= 4, *IQR *= 3,5). For a comprehensive summary of the descriptive results, see Table S4 in the Supplemental Information.

None of the background characteristics significantly affected return of results preferences. Desire to receive results was positively associated with preference for all return of results categories (see Table 5). The strongest associations were found with actionable results that can be immediately used to improve one's health and those that are classified as abnormal and possibly relevant to health. Complete access to all information that is recorded as a result of the reuse of data was associated least strongly.

**Table 5.** Spearman rank-order correlations (*ρ*) between preference for receiving results from reuse and importance of return of results categories.

Variable		1. I want to receive results from reuse of my data that are relevant to my personal health.	2. I want to receive actionable results that can be used immediately to improve my health.	3. I want to receive results that are flagged as abnormal and that are thought to be possibly relevant to health and care.	4. I want to receive all results classified as abnormal regardless of possible relevance to my health.	5. I want to receive information that exposes a potentially dangerous genetic mutation or reproductive risk.	6. I want to have complete access to all information that is recorded as a result of the reuse of my data.
**1. I want to receive results from reuse of my data that are relevant to my personal health.**							
	*ρ*	1	0.448	0.407	0.266	0.335	0.272
	*P* value	—^a^	<.001	<.001	<.001	.687	<.001
**2. I want to receive actionable results that can be used immediately to improve my health.**							
	*ρ*	0.448	1	0.485	0.250	0.366	0.247
	*P* value	<.001	—^a^	<.001	<.001	<.001	<.001
**3. I want to receive results that are flagged as abnormal and that are thought to be possibly relevant to health and care**.							
	*ρ*	0.407	0.485	1	0.476	0.434	0.367
	*P* value	<.001	<.001	—^a^	<.001	<.001	<.001
**4. I want to receive all results classified as abnormal regardless of possible relevance to my health.**							
	*ρ*	0.266	0.250	0.476	1	0.369	0.449
	*P* value	<.001	<.001	<.001	—^a^	<.001	<.001
**5. I want to receive information that exposes a potentially dangerous genetic mutation or reproductive risk.**							
	*ρ*	0.335	0.366	0.434	0.369	1	0.362
	*P* value	.687	<.001	<.001	<.001	—^a^	<.001
**6. I want to have complete access to all information that is recorded as a result of the reuse of my data.**							
	*ρ*	0.272	0.247	0.367	0.449	0.362	1
	*P* value	<.001	<.001	<.001	<.001	<.001	—^a^

^a^ Not applicable.

Desire for re-consent (*ρ *=* *0.035, *P *= .299, *n *= 877), opt out (*ρ *=* *−0.014, *P *= .688, *n *= 855) or a reuse approval committee (*ρ *=* *0.018, *P *= .607, *n *= 852) was not associated with preference for return of results. Re-consent was, however, positively associated with return of results classified as abnormal (*ρ *=* *0.108, *P *= .001, *n *= 861) and complete access to all information recorded (*ρ *=* *0.132, *P *< .001, *n *= 859). Returning genetic information about a potentially dangerous genetic mutation or reproductive risk was also associated positively with preference for a reuse approval committee (*ρ *=* *0.101, *P *= .004, *n *= 832).

### Oversight and sanctions for misuse when reusing health data

A total of 78.6% (*Md *= 5, *IQR *= 5,5) of respondents regarded oversight of misuse of health data in research institutions where researchers are employed as *highly important*. The capacity to impose penalties on researchers was also seen as *highly important* by 64.1% (*Md *= 5, *IQR *= 4,5). Additionally, 99.4% (*Mo *= 1) of the respondents concurred that processing data negligently as well as noncompliance with established rules and procedures constitutes misuse of health data reuse. Attempts by researchers to retrieve someone's identity using their health data were viewed as data misuse by 90.6% of respondents (*Mo *= 1). Data reuse for research that does not directly further scientific or societal purposes was perceived as misuse by 83.4% of respondents (*Mo *= 1), and 79.3% (*Mo *= 1) of respondents regarded misuse as data reuse by new research projects without, or not in accordance with, their permission. Finally, 70.9% of respondents (*Mo *= 1) considered it to be misuse when commercial companies reused their data for scientific research.

The most accurate definition of misuse of health data reuse according to respondents was processing data negligently as well as noncompliance with established rules and procedures (35.6%, *Mo *= 1). The second most accurate definition was attempts by researchers to retrieve someone's identity using their health data (20.4%). See Table S5 in the Supplemental Information for a detailed overview of the descriptive results.

Which definition of misusing health data reuse was considered most appropriate was significantly associated with preference for re-consent (*χ*^2^_20_ (*n = *889) = 74.7, *P *< .001, *φ*_c_*_ _* = 0.145), opt out (*χ*^2^_20_ (*n = *868) = 33.4, *P *= .031, *φ*_c_*_ _* = 0.098) and a reuse approval committee (*χ*^2^_20_ (*n = *862) = 41.4, *P *= .003, *φ*_c_*_ _* = 0.110). Those who preferred re-consent (30.8%), an opt-out system (34.6%) and a reuse approval committee (35.6%) saw negligent data processing and noncompliance with established rules and procedures as the most accurate definition.

The importance of oversight of data misuse in research institutions was positively associated with favouring re-consent (*ρ *=* *0.151, *P *< .001, *n *= 891) and an opt-out system (*ρ *=* *0.073, *P *= .031, *n *= 869). Only preference for re-consent was associated with the ability to impose penalties on researchers (*ρ *=* *0.177, *P *< .001, *n *= 887).

## Discussion

### Principal findings

This study revealed that only 1.4% of the respondents opposed the reuse of health data for scientific research altogether. Almost 70% preferred broad consent, defined as giving consent to a broad range of research questions. Still, a substantial minority of around 30% wanted to consent to specific research questions. Respondents were in remarkable disagreement about whether re-consent before new reuse, an opt-out system to object to reuse and an independent committee for approving if new research could reuse health data were most desirable. Most people thought it important to be informed about health data reuse and its scientific results in general. Providing information by newsletters via email containing short summaries and updates about the research using their data and a digital profile allowing people to tailor communication to their personal preferences as well as enabling interaction with researchers and research participants were greatly favoured. Furthermore, an overwhelming majority of respondents wanted research results from data reuse to be returned to them. Receiving actionable results that can be used immediately to improve one's health, as well as results that are flagged as abnormal and that are thought to be possibly relevant to health and care, and return of information that exposes a potentially dangerous genetic mutation or reproductive risk were regarded as important. Institutional oversight of data misuse by researchers and possibilities to impose sanctions were seen as crucial. Respondents characterized data misuse primarily as negligent data processing and noncompliance with established rules and procedures.

### Comparison with the literature

#### Communication and interaction

Our findings show a strong willingness to share and reuse health data for scientific purposes, which is facilitated by the adaptable approach provided by the flexibility provided by the elements of dynamic consent.^22,28^ Receiving email newsletters with summaries and updates and a digital profile for interaction on a communication platform were strongly preferred. These findings support the interactive model for research participation, which is the foundation of dynamic consent.^22,28,38,56^ Our findings also support previous research showing that people prefer more interactive forms of information, communication and engagement,^30,38^ in particular pointing to the relevance of engaging data practices, enhanced feedback and active participation.^34^ The potential for dynamic consent to foster interactive dialogue and improve communication between participants and researchers is supported. Enabling such communication in a dynamic consent interface allows for the establishment of long-term interactive partnerships, moving research participation beyond passive participation.^28,38^

For large-scale sharing and reuse of health data, our findings support previous research that demonstrates that preferences for obtaining consent and providing information are intertwined elements of participation.^19,42,46,57^ Regardless of support for specific forms of approving health data reuse, the emphasis on facilitating more and better communication with participants stands out. This stresses the importance of dynamic consent in allowing participants to maintain control over how their data are used and for what purposes.^27,30,58^

Our findings point out that more frequent and diverse modes of communication are required. Improving information and communication is especially important as a stepping stone to direct involvement. However, it remains difficult to demonstrate reciprocity, commitment, transparency and veracity in order to foster long-term relationships between participants and researchers.^28,38^ Furthermore, dynamic consent has yet to be developed further in order to facilitate open communication and the incorporation of participant feedback into research.^42,59^ As a result, mechanisms enabling the gathering and incorporation of feedback by data-contributing participants should be developed for dynamic consent interfaces as part of the governance of health data sharing.

The need to give participants the ability to choose how they want to be addressed, informed and involved based on their own preferences was supported. Communication and interaction should thus not simply be enabled; rather, dynamic consent should allow research participants to interface with the research as they see fit. Notably, our findings do also support previous research emphasizing the importance of retaining passive and paper-based forms of participation.^17,38^

#### Consent and authorization

People preferred to give their consent to a broad range of research questions rather than specific research questions. Furthermore, while most people strongly favoured re-consent, a sizable group of people opposed it. Simultaneously, an opt-out system or an independent data reuse committee received more support and less opposition. This supports the idea that specific re-consent should be reconsidered as the only or primary consent option within a dynamic consent interface for data reuse by new research. Instead, our findings indicate that development of alternatives for authorization of data reuse beyond specific re-consent should be taken seriously. One way to do this is by offering meaningful alternatives via dynamic consent.^22,56^ Our findings support prior research showing that participants favour the possibility of capturing and managing evolving consent preferences over time.^30,35^ However, some have argued that focusing on re-consent is expensive and time-consuming and may cause consent fatigue. The latter critique has also been discussed in connection with dynamic consent, yet there is no empirical evidence indicating consent fatigue.^17,22,36,42^ This relates to the debate between specific re-consent and broad consent in the context of biobanking.^17,18,24,37,60^ Rather, our findings suggest that alternatives like broad consent should be embraced, especially when due regard is given to conditions safeguarding trust in the specific research institutions that are involved. People should however remain able to choose between various types of consent for reuse of scientific health data rather than completely replacing specific re-consent. For reuse of health care data, however, broad consent should be approached with greater caution.

There is considerable agreement and overlap between those who favour tighter, more detailed personal control over approval for data reuse^19,40,58,61^ and those who are inclined to give broader approval. Both groups agree that they would like more information about reuse, its outcomes and the results that are returned. Additionally, the assertion that the appeal of control has more to do with support for the idea of control than its actual exercise^19,40,57^ is supported by the strong relation between consent and a desire for more and better communication. This is consistent with the recent proposal for a new Data Governance Act (DGA, EU 2022/868) in the European Union. The DGA aims to add a uniform European data altruism consent for data reuse for general interest purposes, such as scientific research, in order to provide greater transparency and choice for data subjects.^32^ Whereas altruism is often mentioned as an important motivation for sharing data and participating in health research,^1,62^ our results indicate that data-contributing participants expect and want something in return for their participation in scientific health research as well. Participants want information about how their data are used, by whom and for which purposes, as well as relevant, actionable results that could improve their own health.

The DGA also encourages data cooperatives to establish terms and conditions prior to consent, improving informed choice and representing the interests of data subjects. The need for such regulatory innovations is supported by this study. Moreover, the importance of empowerment of patients and research participants underlying the DGA is supported.^32,38^

#### Return of results and information

The vast majority of people desired return of results from research for which their data are used. This supports earlier evidence that research participants have a strong desire to learn about such results from genetic testing and studies. In addition, our findings corroborate that participants see return of results as a crucial component of being informed about data sharing as part of dynamic consent.^20,22,33,43^ Our findings support the need for granularity in choosing and selecting the results that should be disclosed.^33^ People strongly favour return of results that are meaningful and that may have an impact on their own health and care or that of their children. This emphasizes the necessity of health care professionals that can explain utility to participants to improve understanding of results, as opposed to simply wanting to receive either all or no results.^33,63,64^ The findings support the recommendation that interpretation should be aided by providing clear examples that are relevant to participants’ individual health situations.^33,44,64^ Thus, prevention of information overload and maximizing understanding are paramount.^38,45,48^

#### Approval and oversight

People preferred experts rather than patients and citizens to serve as members of an independent data reuse committee. Those who supported broad consent preferred expert members to a greater extent than did proponents of specific consent, just like proponents of an opt-out system and a data reuse committee. As this survey was conducted in a university hospital's patient panel, this points to the role of trust in professionals and experts in institutions and organizations that people are familiar with in the context of large-scale sharing and reuse of health data.^21,38,40^ Also, these findings nuance concerns about delegating control and oversight responsibilities to participants instead of committed professionals in dynamic consent.^17,19,60^ Institutional monitoring and oversight of data misuse and abilities to impose penalties were also seen as crucial. This is in line with research pointing out the importance of having review systems in place.^46^ Findings demonstrate the importance of both technical and policy measures to protect security and privacy, regardless of how respondents want to approve data reuse, receive information or have results returned. Implementing such measures, however, represents a significant challenge both technologically and in terms of how participants experience technological solutions.^20,56,58,61^

#### Accommodating plurality

Findings show that different options should be available when designing a dynamic consent interface for health data reuse. This demonstrates that there are multiple approaches to designing dynamic consent. Additionally, context-sensitive, grounded implementation of dynamic consent is crucial for enabling legitimate, sustainable authorization of data reuse.^21,28,43,57^ However, specific patterns in people's preferences were identified. For instance, whereas broad consent was strongly associated with an independent data reuse committee, specific consent was strongly associated with re-consent. However, creating dynamic consent interfaces should not be a rigid and uniform process. The benefit of dynamism for a participation interface is that users can customize participation components to their own preferences, exercising their individual rights as data providers. By providing a variety of options for research participation, we found that dynamic consent does in fact engage a larger group of research participants with a variety of perspectives, interests and preferences.^22^ Nevertheless, the challenges involved in accommodating specific population groups are not to be underestimated when developing dynamic consent interfaces in practice.^45^

### Strengths and limitations

Although this survey study of dynamic consent is one of the first quantitative studies on the topic, we must recognize that its purposive sampling among members of the University Medical Center Utrecht's patient panel limits the generalizability of the findings. Distribution of the questionnaire among the patient panel affects the representativeness of the sample in relation to the broader population of potential users of a dynamic consent interface in the context of health data reuse for scientific research. Respondents were relatively old, mostly identified as patients and were relatively highly educated. In particular, the high level of education warrants caution since this has likely impacted the results in a more positive way than would otherwise be the case. We sought to accommodate these discrepancies by stratifying the results using the most important demographic characteristics (age, education level and gender) as well as testing for significant relations between these characteristics and the main dependent variables. Whereas differences in age impacted results, this was in line with the expected effect of age on patients’ and public preferences in the context of health research. Moreover, it should be noted that this survey study took place during the COVID-19 pandemic. Although the survey was distributed during the final months of COVID-19 restrictions in the Netherlands, COVID-19 has likely contributed to the fostering of an atmosphere in which people were both more conducive and more opposed to the employment of innovative digital tools in the context of health and research such as dynamic consent. At the same time, our results did not indicate a significant departure from established insights and preferences for dynamic consent.

Strengths of this survey were its questionnaire-based, quantitative approach. This allowed us to coherently inquire about respondents’ preferences for the different elements featured in dynamic consent interfaces to establish their interrelations. Moreover, we ascertained the different elements of dynamic consent in a hypothetical scenario in which previously gathered types of health data would be reused for scientific research. Using a hypothetical scenario, though portraying a simplified, decontextualized situation, allows forestalling the possible confounding effect of specific factors such as types of data used, specific parties involved, previously established relationships and expectations revolving around possible outcomes. In the context of dynamic consent, this approach contributes to the existing body of evidence that mostly relies on qualitative case studies. This study adds to the existing body of literature by going beyond the primary context of biobanking to establish insights into dynamic consent in the context of large-scale health data sharing and reuse.

Still, we recognize the need to bridge both decontextualized quantitative and contextualized qualitative approaches. Future research into dynamic consent would benefit from conducting a contextualized survey inquiry into patient and public preferences. It would be valuable to survey people's preferences for development of a dynamic consent interface within a demarcated, clearly communicated case of health data research before it is articulated and put to use. Doing so would allow situated establishment of people's preferences given contextual circumstances.

## Conclusion

Dynamic consent for large-scale sharing and reuse of health data is more than just a potential solution for asking consent by means of a digital interface. Dynamic consent maintains a promising approach to facilitate participation and interaction with health data research. This study has indicated that enabling abilities for research participants contributing data to obtain adequate, tailored information and insights about research practices increases their sense of control in connection to making consent decisions. Transparent, meaningful communication and return of relevant results to participants are important considerations to take into account when developing dynamic consent interfaces for large-scale health data reuse for scientific research. Facilitating the ability to make such decisions and exert influence allows research participants to control how they participate in large-scale health data research within specific contexts. Dynamic consent attends to this need by focusing on engaged communication and information provision practices in relation to personalized consent options. Last but not least, this emphasizes the importance of collective control by establishing a strong and solid governance architecture as well, in which dynamic consent features as a useful approach.

## Supplemental Material

sj-docx-1-dhj-10.1177_20552076231190997 - Supplemental material for Dynamic consent, communication and return of results in large-scale health data reuse: Survey of public preferences Click here for additional data file. Supplemental material, sj-docx-1-dhj-10.1177_20552076231190997 for Dynamic consent, communication and return of results in large-scale health data reuse: Survey of public preferences by Sam HA Muller, Ghislaine JMW van Thiel, Menno Mostert and Johannes JM van Delden in DIGITAL HEALTH

sj-docx-2-dhj-10.1177_20552076231190997 - Supplemental material for Dynamic consent, communication and return of results in large-scale health data reuse: Survey of public preferences Click here for additional data file. Supplemental material, sj-docx-2-dhj-10.1177_20552076231190997 for Dynamic consent, communication and return of results in large-scale health data reuse: Survey of public preferences by Sam HA Muller, Ghislaine JMW van Thiel, Menno Mostert and Johannes JM van Delden in DIGITAL HEALTH

## References

[1] Kalkman S Mostert M Udo-Beauvisage N , et al. Responsible data sharing in a big data-driven translational research platform: lessons learned. BMC Med Inform Decis Mak 2019; 19: 283. 31888593 10.1186/s12911-019-1001-y PMC6936121

[2] Boers SN van Delden JJM Bredenoord AL . Broad consent is consent for governance. Am J Bioeth 2015; 15: 53–55. 10.1080/15265161.2015.1062165 26305756

[3] Kaye J Terry SF Juengst E , et al. Including all voices in international data-sharing governance. Hum Genomics 2018; 12: 13. 29514717 10.1186/s40246-018-0143-9 PMC5842530

[4] Mostert M Bredenoord AL Biesaart MCIH , et al. Big data in medical research and EU data protection law: challenges to the consent or anonymise approach. Eur J Hum Genet 2016; 24: 956–960. 26554881 10.1038/ejhg.2015.239 PMC5070890

[5] Muller SHA Kalkman S van Thiel GJMW , et al. The social licence for data-intensive health research: towards co-creation, public value and trust. BMC Med Ethics 2021; 22: 110. 34376204 10.1186/s12910-021-00677-5 PMC8353823

[6] Shabani M Dove ES Murtagh M , et al. Oversight of genomic data sharing: what roles for ethics and data access committees? Biopreservation Biobanking 2017; 15: 469–474. 28836815 10.1089/bio.2017.0045

[7] Ruyter KW LÕuk K Jorqui M , et al. From research exemption to research norm: recognising an alternative to consent for large scale biobank research. Med Law Int 2010; 10: 287–313.

[8] Sethi N Laurie GT . Delivering proportionate governance in the era of eHealth: making linkage and privacy work together. Med Law Int 2013; 13: 168–204. 24634569 10.1177/0968533213508974 PMC3952593

[9] Prainsack B Buyx A . A solidarity-based approach to the governance of research biobanks. Med Law Rev 2013; 21: 71–91. 23325780 10.1093/medlaw/fws040

[10] Mittelstadt BD Floridi L . The ethics of big data: current and foreseeable issues in biomedical contexts. Sci Eng Ethics 2016; 22: 303–341. 26002496 10.1007/s11948-015-9652-2

[11] Sheehan M Thompson R Fistein J , et al. Authority and the future of consent in population-level biomedical research. Public Health Ethics 2019; 12: 225–236. 32082417 10.1093/phe/phz015 PMC7020771

[12] Dove ES Townend D Knoppers BM . Data protection and consent to biomedical research: a step forward? Lancet 2014; 384: 855. 25209486 10.1016/S0140-6736(14)61488-4

[13] Laurie G Postan E . Rhetoric or reality: what is the legal status of the consent form in health-related research? Med Law Rev 2013; 21: 371–414. 23055572 10.1093/medlaw/fws031 PMC3746797

[14] Caulfield T . Biobanks and blanket consent: the proper place of the public good and public perception rationales. Kings Law J 2007; 18: 209–226.

[15] Ploug T Holm S . Meta consent—a flexible solution to the problem of secondary use of health data. Bioethics 2016; 30: 721–732. 27628305 10.1111/bioe.12286 PMC5108479

[16] Bunnik EM Janssens ACJW Schermer MHN . A tiered-layered-staged model for informed consent in personal genome testing. Eur J Hum Genet 2013; 21: 596–601. 23169494 10.1038/ejhg.2012.237 PMC3658183

[17] Steinsbekk KS Kare Myskja B Solberg B. Broad consent versus dynamic consent in biobank research: is passive participation an ethical problem. Eur J Hum Genet 2013; 21: 897–902. 23299918 10.1038/ejhg.2012.282 PMC3746258

[18] Stein DT Terry SF . Reforming biobank consent policy: a necessary move away from broad consent toward dynamic consent. Genet Test Mol Biomark 2013; 17: 855–856. 10.1089/gtmb.2013.1550 24283583

[19] Teare HJA Prictor M Kaye J . Reflections on dynamic consent in biomedical research: the story so far. Eur J Hum Genet 2021; 29: 649–656. 33249421 10.1038/s41431-020-00771-z PMC7695991

[20] Thiel DB Platt J Platt T , et al. Testing an online, dynamic consent portal for large population biobank research. Public Health Genomics 2015; 18: 26–39. 25359560 10.1159/000366128 PMC4289420

[21] Williams H Spencer K Sanders C , et al. Dynamic consent: a possible solution to improve patient confidence and trust in how electronic patient records are used in medical research. JMIR Med Inform 2015; 3: e3. 25586934 10.2196/medinform.3525 PMC4319083

[22] Kaye J Whitley EA Lund D , et al. Dynamic consent: a patient interface for twenty-first century research networks. Eur J Hum Genet 2015; 23: 141–146. 24801761 10.1038/ejhg.2014.71 PMC4130658

[23] Budin-Ljøsne I Teare HJA Kaye J , et al. Dynamic consent: a potential solution to some of the challenges of modern biomedical research. BMC Med Ethics 2017; 18: 1–10. 28122615 10.1186/s12910-016-0162-9 PMC5264333

[24] Sheehan M . Broad consent is informed consent. Br Med J 2011; 343: 1. 10.1136/bmj.d6900 22046007

[25] Kaye J . The tension between data sharing and the protection of privacy in genomics research. Annu Rev Genomics Hum Genet 2012; 13: 415–431. 22404490 10.1146/annurev-genom-082410-101454 PMC4337968

[26] Joly Y Dalpé G So D , et al. Fair shares and sharing fairly: a survey of public views on open science, informed consent and participatory research in biobanking. PLOS ONE 2015; 10: e0129893. 26154134 10.1371/journal.pone.0129893 PMC4495996

[27] Muller SHA van Thiel GJMW Vrana M , et al. Patients’ and publics’ preferences for data-intensive health research governance: survey study. JMIR Hum Factors 2022; 9: e36797. 36069794 10.2196/36797 PMC9494211

[28] Kaye J Curren L Anderson N , et al. From patients to partners: participant-centric initiatives in biomedical research. Nat Rev Genet 2012; 13: 371–376. 22473380 10.1038/nrg3218 PMC3806497

[29] Prictor M Lewis MA Newson AJ , et al. Dynamic consent: an evaluation and reporting framework. J Empir Res Hum Res Ethics 2020; 15: 175–186. 31729900 10.1177/1556264619887073

[30] Teare HJ Morrison M Whitley EA , et al. Towards ‘Engagement 2.0’: insights from a study of dynamic consent with biobank participants. Digit Health 2015; 1: 1–13. 10.1177/2055207615605644 PMC6001239 29942545

[31] Hafen E. Personal data cooperatives—a new data governance framework for data donations and precision health. In: Krutzinna J Floridi L (eds) The ethics of medical data donation. Cham: Springer International Publishing, 2019, pp.141–149. 32091856

[32] Shabani M . The Data Governance Act and the EU’s move towards facilitating data sharing. Mol Syst Biol 2021; 17: e10229. 33755313 10.15252/msb.202110229 PMC7985992

[33] Christensen KD Savage SK Huntington NL , et al. Preferences for the return of individual results from research on pediatric biobank samples. J Empir Res Hum Res Ethics 2017; 12: 97–106. 28421887 10.1177/1556264617697839 PMC5407299

[34] Schuler Scott A. Dynamic consent: a mechanism for engagement. University of Oxford, https://ora.ox.ac.uk/objects/uuid:a671615d-ebed-4a7e-987a-481167b1056f (2022, accessed 26 May 2023).

[35] Pacyna JE McCormick JB Olson JE , et al. Assessing the stability of biobank donor preferences regarding sample use: evidence supporting the value of dynamic consent. Eur J Hum Genet 2020; 28: 1168–1177. 32327712 10.1038/s41431-020-0625-9 PMC7608348

[36] Prictor M Teare HJA Kaye J. Equitable participation in biobanks: the risks and benefits of a “dynamic consent” approach. Front Public Health 2018; 6: 253. 30234093 10.3389/fpubh.2018.00253 PMC6133951

[37] Soulier A . Reconsidering dynamic consent in biobanking: ethical and political consequences of transforming research participants into ICT users. IEEE Technol Soc Mag 2019; 38: 62–70.

[38] De Sutter E Borry P Geerts D , et al. Personalized and long-term electronic informed consent in clinical research: stakeholder views. BMC Med Ethics 2021; 22: 108. 34332572 10.1186/s12910-021-00675-7 PMC8325412

[39] Dixon WG Spencer K Williams H , et al. A dynamic model of patient consent to sharing of medical record data. Br Med J 2014; 348: g1294. 24500352 10.1136/bmj.g1294

[40] Spencer K Sanders C Whitley EA , et al. Patient perspectives on sharing anonymized personal health data using a digital system for dynamic consent and research feedback: a qualitative study. J Med Internet Res 2016; 18: e66. 27083521 10.2196/jmir.5011 PMC4851723

[41] Wallace SE Miola J . Adding dynamic consent to a longitudinal cohort study: a qualitative study of EXCEED participant perspectives. BMC Med Ethics 2021; 22: 12. 33563268 10.1186/s12910-021-00583-w PMC7874652

[42] Mascalzoni D Melotti R Pattaro C , et al. Ten years of dynamic consent in the CHRIS study: informed consent as a dynamic process. Eur J Hum Genet 2022; 30: 1391–1397. 36064788 10.1038/s41431-022-01160-4 PMC9441838

[43] Haas MA Teare H Prictor M , et al. ‘CTRL’: an online, dynamic consent and participant engagement platform working towards solving the complexities of consent in genomic research. Eur J Hum Genet 2021; 29: 687–698. 33408362 10.1038/s41431-020-00782-w PMC8115139

[44] Sundby A Boolsen MW Burgdorf KS , et al. The preferences of potential stakeholders in psychiatric genomic research regarding consent procedures and information delivery. Eur Psychiatry 2019; 55: 29–35. 30384109 10.1016/j.eurpsy.2018.09.005

[45] De Sutter E Zaçe D Boccia S , et al. Implementation of electronic informed consent in biomedical research and stakeholders’ perspectives: systematic review. J Med Internet Res 2020; 22: e19129. 33030440 10.2196/19129 PMC7582148

[46] Johansson JV Bentzen HB Shah N , et al. Preferences of the public for sharing health data: discrete choice experiment. JMIR Med Inform 2021; 9: e29614. 36260402 10.2196/29614 PMC8406119

[47] Sharma A Minh Duc NT Luu Lam Thang T , et al. A consensus-based Checklist for Reporting of Survey Studies (CROSS). J Gen Intern Med 2021; 36: 3179–3187. 33886027 10.1007/s11606-021-06737-1 PMC8481359

[48] Dankar FK Gergely M Malin B , et al. Dynamic-informed consent: a potential solution for ethical dilemmas in population sequencing initiatives. Comput Struct Biotechnol J 2020; 18: 913–921. 32346464 10.1016/j.csbj.2020.03.027 PMC7182686

[49] Siegel S Castellan NJ . Nonparametric statistics for the behavioral sciences. Boston, MA: McGraw-Hill, 1988.

[50] Jamieson S . Likert scales: how to (ab)use them. Med Educ 2004; 38: 1217–1218. 15566531 10.1111/j.1365-2929.2004.02012.x

[51] Pett MA . Nonparametric statistics for health care research: statistics for small samples and unusual distributions. 2nd Edn. Thousand Oaks, CA: Sage Publications Inc, 2016.

[52] Knapp TR . Treating ordinal scales as interval scales. Nurs Res 1990; 39: 121–123. 2315066

[53] Sullivan GM Artino AR . Analyzing and interpreting data from Likert-type scales. J Grad Med Educ 2013; 5: 541–542. 24454995 10.4300/JGME-5-4-18 PMC3886444

[54] Tabachnick BG Fidell LS . Using multivariate statistics. 6 th Edn. Boston, MA: Pearson, 2013.

[55] Leeuw E Hox J. Missing data. In: Encyclopedia of survey research methods. Thousand Oaks, CA: Sage Publications, Inc., 2008, pp. 468–471.

[56] Aziz A F M Yusof M , et al. Can dynamic consent facilitate the protection of biomedical big data in biobanking in Malaysia? Asian Bioeth Rev 2019; 11: 209–222. 33717312 10.1007/s41649-019-00086-2 PMC7747242

[57] Teare HJA Hogg J Kaye J , et al. The RUDY study: using digital technologies to enable a research partnership. Eur J Hum Genet 2017; 25: 816–822. 28443622 10.1038/ejhg.2017.57 PMC5520069

[58] Coathup V Teare HJA Minari J , et al. Using digital technologies to engage with medical research: views of myotonic dystrophy patients in Japan. BMC Med Ethics 2016; 17: 51–51. 27553007 10.1186/s12910-016-0132-2 PMC4995774

[59] Mascalzoni D Hicks A Pramstaller PP . Consenting in population genomics as an open communication process. Stud Ethics Law Technol 2009; 3: 1.

[60] Steinsbekk K Solberg B . Biobank consent models: are we moving toward increased participant engagement in biobanking? J Biorepository Sci Appl Med 2015; 3: 23–23.

[61] Despotou G Evans J Nash W , et al. Evaluation of patient perception towards dynamic health data sharing using blockchain based digital consent with the Dovetail digital consent application: a cross sectional exploratory study. Digit Health 2020; 6: 1–11. 10.1177/2055207620924949 PMC7223864 32435503

[62] Shabani M Bezuidenhout L Borry P . Attitudes of research participants and the general public towards genomic data sharing: a systematic literature review. Expert Rev Mol Diagn 2014; 14: 1053–1065. 25260013 10.1586/14737159.2014.961917

[63] Sanderson SC Linderman MD Suckiel SA , et al. Motivations, concerns and preferences of personal genome sequencing research participants: baseline findings from the HealthSeq project. Eur J Hum Genet 2016; 24: 14–20. 26036856 10.1038/ejhg.2015.118 PMC4795230

[64] Graves KD Sinicrope PS McCormick JB , et al. Public perceptions of disease severity but not actionability correlate with interest in receiving genomic results: nonalignment with current trends in practice. Public Health Genomics 2015; 18: 173–183. 25790929 10.1159/000375479

