# Supplementary material for: Dynamic consent, communication and return of results in large-scale health data reuse: Survey of public preferences
Source: Digit Health. 2023 Aug 16;9:20552076231190997. doi: 10.1177/20552076231190997 (PMC10434987; doi:10.1177/20552076231190997)
Supplement: sj-docx-1-dhj-10.1177_20552076231190997 - Supplemental material for Dynamic consent, communication and return of results in large-scale health data reuse: Survey of public preferences [file sj-docx-1-dhj-10.1177_20552076231190997.docx]

**Supplementary Information 1**

Table S1. Frequencies and descriptive statistics: general attitude and consent for reuse.

| Variable | 1. % Strongly oppose *(n)* | | 2. % Somewhat oppose *(n)* | | 3. % Neutral *(n)* | 4. % Somewhat favour *(n)* | | 5. % Strongly favour *(n)* | *Md* | IQR |
| --- | --- | --- | --- | --- | --- | --- | --- | --- | --- | --- |
| **What do you think of reuse of your health data for sci­entific health research?** | 0.2 *(2)* | | 1.2 *(11)* | | 4.6 *(41)* | 29.4 *(264)* | | 64.6 *(581)* | 5 | 4, 5 |
|  | | 1. % Agree *(n)* | | 2. % Don’t agree *(n)* | | | 3. % I’m not sure *(n)* | | *Mo* | |
| **I want to know the specific research question when consenting to reuse of my health data** | | 61.6 *(548)* | | 32.6 *(290)* | | | 5.7 *(51)* | | 1 | |
| **I want to consent to a broad range of research questions for reuse of my health data** | | 81.9 *(732)* | | 8.7 *(78)* | | | 9.4 *(84)* | | 1 | |
|  | | | | % *(n)* | | | | | *Mo* | |
| ***Which option for giving consent to reuse of your health data do you pre­fer?*** | | | | | | | | | *2* |  |
| **1. I want to know the specific research question when consenting to reuse of my health data** | | | | 30.2 *(272)* | | | | |  | |
| **2. I want to consent to a broad range of research questions for reuse of my health data** | | | | 69.8 *(628)* | | | | |  | |

Table S2. Frequencies and descriptive statistics: modes of approving reuse.

| Variable | | | 1. % Strongly oppose *(n)* | | 2. % Somewhat oppose *(n)* | | 3. % Neutral *(n)* | | | 4. % Somewhat favour *(n)* | | 5. % Strongly favour *(n)* | | *Md* | IQR |
| --- | --- | --- | --- | --- | --- | --- | --- | --- | --- | --- | --- | --- | --- | --- | --- |
| ***How do you want reuse of your health data to be approved?*** | | | | | | | | | | | | | | |  |
| **1. I want to re-consent before new research can reuse my health data** | | | 20.6 *(184)* | | 16.8 *(150)* | | 17.1 *(153)* | | | 19.8 *(177)* | | 25.7 *(230)* | | 3 | 2, 5 |
| **2. I can object to (opt out of) reuse of my health data by new re­search; I will not be asked to re-consent.** | | | 15.1 *(132)* | | 13.2 *(115)* | | 28.1 *(245)* | | | 20.8 *(181)* | | 22.8 *(199)* | | 3 | 2, 4 |
| **3. An independent committee approves whether new research can reuse my health data** | | | 18 *(156)* | | 11.1 *(96)* | | 24.7 *(214)* | | | 21.9 *(190)* | | 24.2 *(210)* | | 3 | 2, 4 |
| ***Who should be part of an independent committee approving health data reuse?*** | | | | | | | | | | | | | | |  |
| **1. Mainly experts like scien­tists, lawyers and ethicists** | | | 9.4 *(81)* | | 6.5 *(56)* | | 14.8 *(128)* | | | 26.4 *(229)* | | 43 *(372)* | | 4 | 3, 5 |
| **2. Next to experts, (represent­atives of) patients and citi­zens** | | | 14.6 *(126)* | | 12.7 *(109)* | | 22.8 *(196)* | | | 22.6 *(195)* | | 27.3 *(235)* | | 3 | 2, 5 |
|  | 1. % Daily *(n)* | 2. % Weekly *(n)* | | 3. % Biweekly *(n)* | 4. % Monthly *(n)* | 5. % Twice a year *(n)* | | | 6. % Annually *(n)* | | 7. % < Less than annually *(n)* | | 8. % I don’t care *(n)* | *Md* | IQR |
| **How often do you want to be asked for con­sent at most?** | 0.9 *(8)* | 2.8 *(25)* | | 2.1 *(19)* | 14.2 *(128)* | 19.4 *(175)* | | 20.9 (*188)* | | | 6.9 *(62)* | | 32.8 *(295)* | 6 | 5, 8 |

Table S3. Frequencies and descriptive statistics: communication and information regarding reuse.

| Variable | 1. % Yes *(n)* | | 2. % No *(n)* | | | 3. % I’m not sure *(n)* | | *Mo* |  |
| --- | --- | --- | --- | --- | --- | --- | --- | --- | --- |
| **I want to be informed about the reuse of my health data** | 71.3 *(643)* | | 23.1 *(208)* | | | 5.7 *(51)* | | 1 |  |
| **I want to be informed about the sci­entific results attained with my health data** | 83.9 *(756)* | | 10 *(90)* | | | 6.1 *(55)* | | 1 |  |
|  | 1. % Strongly oppose *(n)* | 2. % Somewhat oppose *(n)* | | 3. % Neutral *(n)* | 4. % Somewhat favour *(n)* | | 5. % Strongly favour *(n)* | *Md* | IQR |
| ***How do you want to be in­formed when your health data are reused in scien­tific health research?*** | | | | | | | |  |  |
| **1. I want to be informed by means of a website with up-to-date information about research reusing my data.** | 11.9 *(103)* | 11.6 *(101)* | | 26.2 *(227)* | 31.5 *(273)* | | 18.8 *(163)* | 4 | 3, 4 |
| **2. I want to be informed by means of email newsletters containing short summaries and updates about research using my data.** | 4.3 *(38)* | 4.8 *(42)* | | 9 *(79)* | 46.5 *(406)* | | 35.4 *(309)* | 4 | 4, 5 |
| **3. I want to be informed by means of a digital profile allowing me to tailor communication to my personal preferences; this allows me to interact with researchers and research participants** | 2.9 *(25)* | 4.7 *(40)* | | 22.1 *(190)* | 36.9 *(317)* | | 33.5 *(288)* | 4 | 3, 5 |

Table S4. Frequencies and descriptive statistics: return of results.

| Variable | 1. % Strongly disagree *(n)* | 2. % Dis­agree *(n)* | 3. % Nei­ther agree nor disa­gree *(n)* | 4. % Agree *(n)* | 5. % Strongly agree *(n)* | *Md* | IQR |
| --- | --- | --- | --- | --- | --- | --- | --- |
| **Do you want to have re­search results of your data that are relevant to your health re­turned to you?** | 1.7 *(15)* | 1.1 *(10)* | 5.2 *(46)* | 13.2 *(117)* | 78.8 *(697)* | 5 | 5, 5 |
|  | 1. % Highly unim­portant *(n)* | 2. % Fairly un­important *(n)* | 3. % Neither important nor unimportant *(n)* | 4. % Moderately important *(n)* | 5. % Highly im­portant *(n)* | *Md* | IQR |
| ***What is your opin­ion about receiving the following types of results?*** | | | | | |  |  |
| **1. I want to receive actionable results that can be used immediately to improve my health.** | 1.5 *(13*) | 0.6 *(5)* | 3.6 *(32)* | 12.9 *(114)* | 81.4 *(720)* | 5 | 5, 5 |
| **2. I want to receive results that are flagged as ab­normal and that are thought to be possibly relevant to health and care**. | 1.5 *(13)* | 1.1 *(10)* | 8.8 *(77)* | 33.3 *(292)* | 55.3 *(484)* | 5 | 4, 5 |
| **3. I want to receive all results classified as abnormal regardless of possible relevance to my health.** | 4.7 *(41)* | 11.9 *(103)* | 29.3 *(255)* | 28.5 *(248)* | 25.5 *(220)* | 4 | 3, 5 |
| **4. I want to receive genetic information that exposes a potentially dangerous genetic mutation or reproductive risk.** | 5.7 *(49)* | 4.9 *(42)* | 8.8 *(76)* | 21.2 *(183)* | 59.5 *(514)* | 5 | 4, 5 |
| **5. I want to have complete access to all information that is recorded as a result of the reuse of my data.** | 4.2 *(36)* | 7.4 *(64)* | 18.9 *(164)* | 25.2 *(218)* | 44.3 *(384)* | 4 | 3, 5 |

Table S5. Frequencies and descriptive statistics: sanctions and oversight for reuse.

| Variable | 1. % Highly un­important *(n)* | | 2. % Fairly un­im­portant *(n)* | 3. % Neither important nor unimportant *(n)* | | | 4. % Moderately important *(n)* | 5. % Highly important *(n)* | | *Md* | IQR |
| --- | --- | --- | --- | --- | --- | --- | --- | --- | --- | --- | --- |
| **How important do you find oversight of misuse of health data in research institutions where researchers are employed?** | 4 *(36)* | | 1.2 *(11)* | 1.9 *(17)* | | | 14.2 *(128)* | 78.6 *(707)* | | 5 | 5, 5 |
| **How important do you find the capacity to impose penalties on researchers?** | 2.6 *(23)* | | 1.2 *(11)* | 4.6 *(41)* | | | 27.5 *(246)* | 64.1 *(574)* | | 5 | 4, 5 |
|  |  |  | | |  | 1. % Agree *(n)* | | | 2. % Don’t agree *(n)* | *Mo* | |
| ***What do you see as misuse of reusing your health data?*** | | | | | |  | | | |  |  |
| **1. When my data are processed negligently and in noncompliance with established rules and procedures; for example, when my data are not stored safely.** | | | | | | 99.4 *(897)* | | | 0.6 *(5)* | 1 |  |
| **2. When my data are reused for research that does not directly further scien­tific or societal purposes.** | | | | | | 83.4 *(751)* | | | 16.6 *(187)* | 1 |  |
| **3. When my data are reused by new research projects without, or not in accordance with, previously given consent.** | | | | | | 79.3 *(715)* | | | 20.7 *(187)* | 1 |  |
| **4. When my data are reused for scientific research by commercial companies, like pharmaceutical companies or companies developing medical equip­ment.** | | | | | | 70.9 *(638)* | | | 29.1 *(262)* | 1 |  |
| **5. When researchers attempt to retrieve my identity using my health data.** | | | | | | 90.6 *(816)* | | | 9.4 *(85)* | 1 |  |
|  | | | | | | % *(n)* | | |  | *Mo* |  |
| ***Which definition do you find most appropriate?*** | | | | | |  | | |  | 1 |  |
| **1. When my data are processed negligently and in noncompliance with established rules and procedures; for example, when my data are not stored safely.** | | | | | | 35.6 *(319)* | | |  |  |  |
| **2. When my data are reused for research that does not directly further scien­tific or societal purposes.** | | | | | | 9.3 *(83)* | | |  |  |  |
| **3. When my data are reused by new research projects without, or not in accordance with, previously given consent.** | | | | | | 13.0 *(117)* | | |  |  |  |
| **4. When my data are reused for scientific research by commercial companies, like pharmaceutical companies or companies developing medical equip­ment.** | | | | | | 13.8 *(124)* | | |  |  |  |
| **5. When researchers attempt to retrieve my identity using my health data.** | | | | | | 20.4 *(183)* | | |  |  |  |
| **6. None of the above.** | | | | | | 7.9 *(71)* | | |  |  |  |
