# Supplementary material for: Dynamic consent, communication and return of results in large-scale health data reuse: Survey of public preferences
Source: Digit Health. 2023 Aug 16;9:20552076231190997. doi: 10.1177/20552076231190997 (PMC10434987; doi:10.1177/20552076231190997)
Supplement: sj-docx-2-dhj-10.1177_20552076231190997 - Supplemental material for Dynamic consent, communication and return of results in large-scale health data reuse: Survey of public preferences [file sj-docx-2-dhj-10.1177_20552076231190997.docx]

**Supplementary Information 2**

**What is important for you to give consent for reuse of your health data for scientific health research?**

Thank you for your participation in this survey. This survey study is part of a European project called BigData@Heart. The purpose of the project is to create digital opportunities for large-scale research in cardiovascular health through the sharing and collation of health data.

This questionnaire is about giving consent for reuse of health data for scientific research. We would appreciate it if you could give your opinion about this. **However, by participating in this questionnaire, you do not consent to your health data being used in any way whatsoever!** We are only interested in learning about your opinion about this topic.

Health data may include:

- General information such as age and gender
- Data about the disease, the treatment and its effect
- Laboratory results such as blood and urine tests
- The results of imaging tests, such as X-rays, MRI and CT scans
- Data collected via wearables. For example, the sleep rhythm or the number of steps taken are tracked by a smartwatch
- Body tissue/material stored for research, such as DNA or tumour tissue

The researchers who prepared this survey work as researchers at the University Medical Center Utrecht in the Netherlands. For more information about this project, please visit <https://bigdata-heart.eu>. If you have any questions regarding this survey, please contact Sam Muller, MSc. at [smuller4@umcutrecht.nl](mailto:smuller4@umcutrecht.nl).

This survey consists of 21 questions and will take you roughly 12 minutes to fill in. By responding to the survey, you will consent to your answers being used in a scientific research study about preferences for consenting to health data reuse by our team at the University Medical Center Utrecht. Your answers will remain anonymous to the researchers. We aim to publish our findings in a scientific journal. Do you want to receive updates about this study? Please send an e-mail to Sam Muller, MSc. at [smuller4@umcutrecht.nl](mailto:smuller4@umcutrecht.nl). You will be reminded of this opportunity at the end of the questionnaire.

Thank you very much for you participation in this survey study,


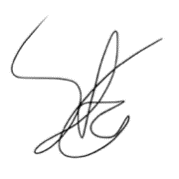


Sam Muller, MSc,

Also on behalf of Prof. dr. Hans van Delden and the University Medical Center Utrecht research team.

Data are being collected in all sorts of ways in the health sector. Maybe you participate in research, but your electronic health record also contains valuable information.

**In this questionnaire, you do not give consent to reuse of your health data.** The following questions concern situations that might take place. In these situations, your consent for reuse of your health data is being asked. In case you give consent, your data will be shared and collated with other people’s health data with the aim of improving treatment and diagnosis. For instance:

You are visiting your GP since you have noticed that you have heart problems. Your GP examines your symptoms and records this information in your electronic health record. If you are being referred to the hospital, information about your complaints, symptoms, the examination and the treatment will be recorded in your health record as well. Whereas this information is recorded in the context of your treatment, they can be of value to research. Researchers would therefore like to reuse your data for health research on cardiovascular disease.

Your health data will be coded when they are being reused for research. This means that your health data will no longer be directly traceable to you. They do not contain any information such as your name or address. Researchers will only receive and use data that have been coded: your identity remains unknown. Your healthcare provider stores the key to your coded health data in a highly secure environment. Nobody will be able to retrieve that your data are used in the research. You can always ask for your data to be deleted if you desire this.

Q1. In general, what do you think of reuse of your health data for scientific health research?

| Strongly oppose |  |  | Neutral |  |  | Strongly favour | I don’t know |
| --- | --- | --- | --- | --- | --- | --- | --- |
| O | O | O | O | O | O | O | O |

**Consent for reuse of your health data**

There are many ways in which your consent for reuse of your health data can be asked. For example, you can be asked to give consent for each research question separately, or you can consent to a broad range of research questions at once. We would like to know which way of giving consent you prefer.

***Specific or broad consent for reuse***

Q2. If I am asked to give consent for reuse of my health data, I want to know for which specific research question my data will be used.

For instance: You are being asked to consent to reuse of your data for research about which complications arise after people have undergone a specific type of operation. In this case, your data may not be used for other research questions than this one.

0 Agree.

0 Don’t agree.

0 I’m not sure.

0 I don’t know.

Could you provide an explanation of your answer?


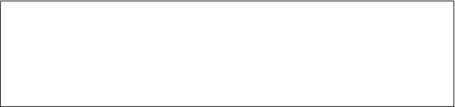


Q3. If I am asked to give consent for reuse of my health data, I want to consent to my data being used for a broad range of research questions.

For instance: Any scientific research that contributes to better treatments and diagnoses of cardiovascular disease. Depending on the research, several of your health data may be used.

0 Agree.

0 Don’t agree.

0 I’m not sure.

0 I don’t know.

Could you provide an explanation of your answer?


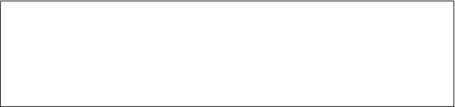


Q4. Which option for giving consent to reuse of your health data do you prefer?

0 I want to know the specific research question when consenting to reuse of my health data.

0 I want to consent to a broad range of research questions for reuse of my health data.

***Ways to ask consent for reuse***

Q5. How do you want to be asked to give consent for reuse of your health data?

For new research that wants to reuse my health data …

1. I want to be asked to *consent again* before new research can reuse my health data.

| Strongly oppose |  |  | Neutral |  |  | Strongly favour | I don’t know |
| --- | --- | --- | --- | --- | --- | --- | --- |
| O | O | O | O | O | O | O | O |

1. I can *object to* reuse of my health data by new research; I will not be asked to consent again.

| Strongly oppose |  |  | Neutral |  |  | Strongly favour | I don’t know |
| --- | --- | --- | --- | --- | --- | --- | --- |
| O | O | O | O | O | O | O | O |

1. An *independent committee* approves whether new research can reuse my health data.

| Strongly oppose |  |  | Neutral |  |  | Strongly favour | I don’t know |
| --- | --- | --- | --- | --- | --- | --- | --- |
| O | O | O | O | O | O | O | O |

1. Other, namely:


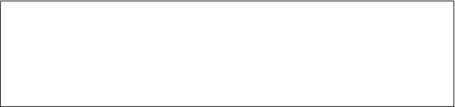


Q6. If an independent committee were to determine whether your health data may be reused for scientific research, who should be part of such a committee?

1. Mainly experts like scientists, lawyers and ethicists.

| Strongly oppose |  |  | Neutral |  |  | Strongly favour | I don’t know |
| --- | --- | --- | --- | --- | --- | --- | --- |
| O | O | O | O | O | O | O | O |

1. Next to experts, (representatives of) *patients and citizens* whose health data are reused.

| Strongly oppose |  |  | Neutral |  |  | Strongly favour | I don’t know |
| --- | --- | --- | --- | --- | --- | --- | --- |
| O | O | O | O | O | O | O | O |

1. Other, namely:


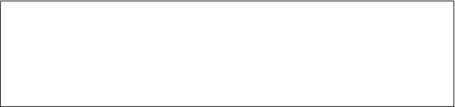


Q7. How often do you want to be asked for con-sent at most?

0 Daily

0 Weekly

0 Twice a week

0 Monthly

0 Twice a year

0 Annually

0 Less than annually

0 I don’t care

**Communication and information regarding reuse**

If your health data are reused, there are multiple ways in which you can receive information about what happens with your data. For instance, which information you would like to receive and how you would like to receive this information. We would like to know what you think about the options presented below for providing you with such information.

***Information about reuse of your health data***

Q8. Do you want to be informed about reuse of your health data?

0 Yes

0 No

0 I don’t know.

***The way in which you receive information about reuse of your health data***

Q9. *How* do you want to be informed when your health data are reused for new scientific health research projects?

1. I want to be informed by means of a *website* with up-to-date information about the funders, goals and questions of the research reusing my data. The website contains updates about research projects that use my health data.

| Strongly oppose |  |  | Neutral |  |  | Strongly favour | I don’t know |
| --- | --- | --- | --- | --- | --- | --- | --- |
| O | O | O | O | O | O | O | O |

1. I want to be informed by means of *email newsletters*. These contain short summaries and updates about research projects using my health data.

| Strongly oppose |  |  | Neutral |  |  | Strongly favour | I don’t know |
| --- | --- | --- | --- | --- | --- | --- | --- |
| O | O | O | O | O | O | O | O |

1. I want to be informed by means of a *digital profile* that allows me to determine which information I want to receive. I can share experiences and questions with other research participants. I can also ask questions and engage in conversations with researchers using my health data.

| Strongly oppose |  |  | Neutral |  |  | Strongly favour | I don’t know |
| --- | --- | --- | --- | --- | --- | --- | --- |
| O | O | O | O | O | O | O | O |

1. Other, namely:


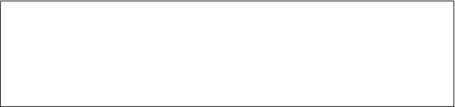


Q10. Do you want to be informed about the *scientific results* obtained with the help of your health data?

0 Yes.

0 No.

0 I don’t know.

**Misuse of your health data**

Without support of patients and citizens for reuse and sharing of health data, scientific research cannot take place. Therefore, it’s important for us to know what you think of misuse of your health data.

Q11. What do you see as misuse if your health data are reused?

1. When my data are processed negligently and in noncompliance with established rules and procedures. For example, when my data are not stored safely.

0 Agree.

0 Disagree.

1. When my data are reused for research that does not directly further scientific or societal purposes. For instance, research that does not directly serve the purpose of improving diagnoses and treatments of disease.

0 Agree.

0 Disagree.

1. When my data are reused by new research projects without, or not in accordance with, previously given consent.

0 Agree.

0 Disagree.

1. When my data are reused for scientific research by commercial companies, like pharmaceutical companies or companies developing medical equipment.

0 Agree.

0 Disagree.

1. When researchers attempt to retrieve my identity using my health data.

0 Agree.

0 Disagree.

1. Other, namely:


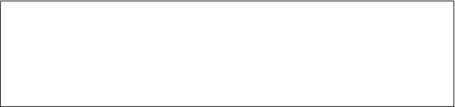


Q12. Which definition do you find most appropriate?

0 When my data are processed negligently and in noncompliance with established rules and procedures; for example, when my data are not stored safely.

0 When my data are reused for research that does not directly further scientific or societal purposes.

0 When my data are reused by new research projects without, or not in accordance with, previously given consent.

0 When my data are reused for scientific research by commercial companies, like pharmaceutical companies or companies developing medical equipment.

0 When researchers attempt to retrieve my identity using my health data.

0 Other, namely:


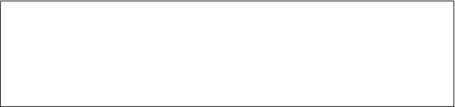


Q13. How important do you find oversight of misuse of health data in research institutions where researchers are employed?

| Highly unimportant |  |  | Neither important nor unimportant |  |  | Highly important | I don’t know |
| --- | --- | --- | --- | --- | --- | --- | --- |
| O | O | O | O | O | O | O | O |

Q14. How important do you find the capacity to impose penalties on researchers?

| Highly unimportant |  |  | Neither important nor unimportant |  |  | Highly important | I don’t know |
| --- | --- | --- | --- | --- | --- | --- | --- |
| O | O | O | O | O | O | O | O |

**Return of results**

If your health data reused for new research, it is possible that information will be found that is relevant to your health. These are known as results. For instance, it might be the case that research reusing your health data finds that you have a strongly increased risk of stroke. It might also be the case that you are carrying a serious disease with you that you might pass on to your children. Since your health data will be coded, in such a case you will always be informed by a physician, not by a researcher.

***Return or no return of results***

Q15. Do you want to have research results of your data that are relevant to your health re¬turned to you?

| Strongly disagree |  |  | Neutral |  |  | Strongly agree | I don’t know |
| --- | --- | --- | --- | --- | --- | --- | --- |
| O | O | O | O | O | O | O | O |

Q16. What is your opinion about receiving the following types of results?

1. Results that are *of direct importance* to my health. For instance, results that might be life-saving, or results about an important health problem. This improves your physician’s ability to make life-saving decisions or improve your treatment.

| Highly unimportant |  |  | Neither important nor unimportant |  |  | Highly important | I don’t know |
| --- | --- | --- | --- | --- | --- | --- | --- |
| O | O | O | O | O | O | O | O |

1. Results that are *flagged as abnormal and that are thought to be possibly relevant* to my health. Your care might be improved with the help of these results. Treatments or prevention of illnesses are possibly still in development or not yet common.

| Highly unimportant |  |  | Neither important nor unimportant |  |  | Highly important | I don’t know |
| --- | --- | --- | --- | --- | --- | --- | --- |
| O | O | O | O | O | O | O | O |

1. *All results classified as abnormal,* regardless of their possible relevance to my health. Based on these results, generally no action is undertaken.

| Highly unimportant |  |  | Neither important nor unimportant |  |  | Highly important | I don’t know |
| --- | --- | --- | --- | --- | --- | --- | --- |
| O | O | O | O | O | O | O | O |

1. *Genetic information* that exposes a potentially dangerous mutation or reproductive risk that does not affect me, but which might affect my children.

| Highly unimportant |  |  | Neither important nor unimportant |  |  | Highly important | I don’t know |
| --- | --- | --- | --- | --- | --- | --- | --- |
| O | O | O | O | O | O | O | O |

1. *Complete access* to all information that is recorded in research projects as a result of the reuse of my data*.*

| Highly unimportant |  |  | Neither important nor unimportant |  |  | Highly important | I don’t know |
| --- | --- | --- | --- | --- | --- | --- | --- |
| O | O | O | O | O | O | O | O |

***Background information***

And finally, please tell us something about yourself (not mandatory)

Q17. What is your gender?

O Female

O Male
O Other
O Prefer not to say

Q18. What is your age category? (In years)

O 18-30

O 31-40
O 41-50

O 51-60

O 61-70
O 71+
O Prefer not to say

Q19. Which country do you live in?

[Drop down menu of nationalities/countries]

Q20. What is your highest level of education?

O Primary school

O Secondary/high school

O Initial vocational education

O Secondary vocational education

O Higher education

O Academic education

O Other

Q21. Do you see yourself as someone with a heart or vascular disease?

O Yes

O No

O Prefer not to say

Is there anything you would like to add to the information in this survey?
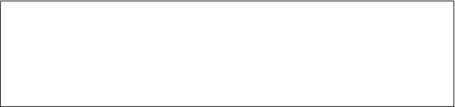


We thank you for taking part in this survey! If you wish to receive updates about this study, please send an e-mail to Sam Muller, MSc. at [smuller4@umcutrecht.nl](mailto:smuller4@umcutrecht.nl).
